# Shifting from Right to Left: The Combined Effect of Elevated CO_2_ and Temperature on Behavioural Lateralization in a Coral Reef Fish

**DOI:** 10.1371/journal.pone.0087969

**Published:** 2014-01-31

**Authors:** Paolo Domenici, Bridie J. M. Allan, Sue-Ann Watson, Mark I. McCormick, Philip L. Munday

**Affiliations:** 1 CNR-IAMC- Istituto per l'Ambiente Marino Costiero, Località Sa Mardini, Torregrande (Oristano) Italy; 2 ARC Centre of Excellence for Coral Reef Studies, James Cook University, Townsville, Queensland, Australia; 3 School of Marine and Tropical Biology, James Cook University, Townsville, Queensland, Australia; University of Sussex, United Kingdom

## Abstract

Recent studies have shown that elevated CO_2_ can affect the behaviour of larval and juvenile fishes. In particular, behavioural lateralization, an expression of brain functional asymmetries, is affected by elevated CO_2_ in both coral reef and temperate fishes. However, the potentially interacting effects of rising temperatures and CO_2_ on lateralization are unknown. Here, we tested the combined effect of near-future elevated-CO_2_ concentrations (930 µatm) and temperature variation on behavioural lateralization of a marine damselfish, *Pomacentrus wardi*. Individuals exposed to one of four treatments (two CO_2_ levels and two temperatures) were observed in a detour test where they made repeated decisions about turning left or right. Individuals exposed to current CO_2_ and ambient temperature levels showed a significant right-turning bias at the population level. This biased was reversed (i.e. to the left side) in fish exposed to the elevated-CO_2_ treatment. Increased temperature attenuated this effect, resulting in lower values of relative lateralization. Consequently, rising temperature and elevated CO_2_ may have different and interactive effects on behavioural lateralization and therefore future studies on the effect of climate change on brain functions need to consider both these critical variables in order to assess the potential consequences for the ecological interactions of marine fishes.

## Introduction

The amount of CO_2_ dissolved in the ocean and the average temperature of the ocean are rising as a result of increasing concentrations of atmospheric CO_2_ [1]. Recent work has shown that increased CO_2_ levels predicted to occur in the ocean by the end of this century can have negative effects on a number of behavioural attributes of marine fishes, such as anti-predator responses [2], [3], sensory performance [4], [5] and lateralization [6], [7]. Behavioural changes induced by elevated CO_2_ can have significant negative consequences for survival in natural coral reef habitats [8] and for a range of other ecological processes, such as habitat selection, timing of settlement and navigation to resting sites [9], [10]. Behavioural changes induced by elevated CO_2_ are reversed by treatment with an antagonist of the GABA-A receptor, suggesting that CO_2_ interferes with neurotransmitter function [11], [12]. While temperature is known to affect the physiological performance and metabolism of coral reef fishes [13], little is known of its effect on behavioural tasks such as lateralization, or the potential interactive effects of elevated CO_2_ and ocean warming on the behaviour of marine organisms.

In fishes and other animals, individuals may show a tendency to turn more often in one direction in a T-maze (detour test), or to use the left or right limb more often when facing a specific task [14]. This behavioural lateralization is an expression of brain asymmetry [14], [15]. The degree of lateralization is likely to reflect a trade-off between the higher performance of lateralized individuals in schooling [16], escape responses [17] and multitasking [18], as a result of their higher cognitive performance [14], compared with the ability of non-lateralized individuals to deal equally well with stimuli or threats from all directions [14]. Lateralization level can also vary according to predation pressure, with individuals from high-predation sites exhibiting stronger lateralization than those from low-predation sites [19]. Studies of fish lateralization behaviour using the detour test have shown that many species tend to have a bias at the population level, although species with non-lateralized populations and a directional bias only at the individual level also occur [15]. Recent work has also shown that behavioural lateralization at the individual level is disrupted by exposure to elevated CO_2_ [7], [20]; however, whether the effects of elevated CO_2_ on lateralization are influenced by water temperature is currently unknown. Projected increases in ocean temperature could interact with elevated CO_2_ levels to further alter the effects on behavioural lateralization. In the present study we test the interactive effects of elevated CO_2_ and temperature variation on individual and population-level lateralization in a coral reef damselfish.

## Materials and Methods

### Ethics statement

Research was carried out under approval of the James Cook University animal ethics committee (permit: A1720) and according to the University's animal ethics guidelines. Fish collections around Lizard Island, Great Barrier Reef were carried with permission of the Great Barrier Reef Parks Authority (permit: G10/33239.1) and Queensland Government Department of Primary Industry and Fisheries (permit: 103256).

### Animals

The study species *Pomacentrus wardi* is small planktivorous damselfish commonly found on Indo-Pacific coral reefs, where it forms extensive aggregations on the reef crest. Settlement-stage larvae of *Pomacentrus wardi* were collected overnight using light traps moored in open water on the western side of Lizard Island (14°40′S, 145°28′E), in the northern Great Barrier Reef, Australia, during October 2012. Juveniles were then maintained in identical rearing tanks at either control (∼405 µatm) or elevated CO_2_ (∼930 µatm) cross-factored with control (∼27°C) or elevated temperature (∼30°C). Control conditions were ambient for the study site at the time of the study and elevated CO_2_ (930 µatm) and temperature (+3°C) treatments matched projected increases by year 2100 [20]. For fish in the elevated temperature treatment group, the temperature was raised by 1°C each 8 hours until the final temperature of ∼29.7°C was reached. This was to avoid any stress associated with rapid temperature increases. Both groups were transferred to 35-L tanks with four treatments: Control- CO_2_/Control-Temperature (C- CO_2_/C-Temp i.e. 396 μatm, 26.7°C), Control- CO_2_/Elevated-Temperature (C-CO2/E-Temp, i.e. 415 µatm, 29.6°C), Elevated- CO_2_/Control-Temperature (E- CO_2_/C-Temp, i.e. 921 µatm, 26.8°C) and Elevated- CO_2_/Elevated-Temperature (E- CO_2_/E-Temp, i.e. 935 µatm, 29.8°C) (Table 1) for a period of seven days. Previous work has shown that the behavioural effects of elevated CO_2_ are manifest within 4 days of exposure to relevant CO_2_ treatments, and that longer durations of exposure do not alter behavioural responses [8], therefore larvae were maintained in the CO_2_ treatments for seven consecutive days. Each tank contained 25 fish. Fish were fed ab libitum with Artemia sp. A 12 hour light and 12 hour dark regime was used.

**Table 1:** Mean (±SE) seawater parameters in the experimental system.

CO_2_ treatment	Temperature treatment	Temperature (°C)	Salinity (ppt)	pH_NBS_	Total alkalinity (µmol.kg^−1^ SW)	*p*CO_2_ (µatm)
Control-CO_2_	Control temperature	26.7 (±0.1)	35.2	8.18 (±0.01)	2274 (±6)	396 (±8)
Control-CO_2_	Elevated temperature	29.6 (±0.1)	35.2	8.17 (±0.01)	2274 (±6)	415 (±9)
Elevated-CO_2_	Control temperature	26.8 (±0.1)	35.2	7.87 (±0.01)	2257 (±6)	921 (±19)
Elevated-CO_2_	Elevated temperature	29.8 (±0.1)	35.2	7.87 (±0.01)	2257 (±6)	935 (±19)

Temperature, pH salinity, and total alkalinity (TA) were measured directly. *p*CO_2_ was estimated from these parameters using CO2SYS.

### CO_2_ and temperature treatments

Tanks were heated with 300-watt bar heaters. Tanks were insulated with Insulbreak to ensure stability of the chosen temperatures of 26.7°C and 29.7°C. CO_2_ treatments were maintained by CO_2_ dosing to a set pH_NBS_. Seawater was pumped from the ocean into 60 L sumps where it was diffused with ambient air (control) or CO_2_ to achieve a pH of 7.87. The pH value was selected to achieve the approximate CO_2_ conditions required, based on preliminary observations of total alkalinity, salinity and temperature of seawater at Lizard Island. A pH-controller (Aqua Medic, Germany) was attached to the CO_2_ treatment sump to maintain pH at the desired level. A solenoid injected a slow stream of CO_2_ into a powerhead at the bottom of the sump whenever the pH of the seawater rose above the set point. Equilibrated seawater from each sump was supplied at a rate of ∼720 ml.min^−1^ to four replicate 35-L aquaria, each housing a small group of larval fish. Temperature and pH_NBS_ of each aquarium was measured daily using a pH meter (HQ40d, Hach, Colorado, USA) calibrated with fresh buffers and a temperature probe (C22, Comark, Norwich, UK). Total alkalinity of seawater was estimated by Gran titration (888 Titrando, Metrohm, Switzerland) from water samples taken twice weekly from control and treatment tanks. Alkalinity standardizations achieved accuracy within 1% of certified reference material from Dr. A. Dickson (Scripps Institution of Oceanography). Seawater p CO_2_ was calculated from seawater parameters in the program CO2SYS [21] using the constants of Mehrbach et al. [22], refit by Dickson and Millero [23]. Seawater parameters are shown in Table 1.

### Lateralization test

**Figure 1:**
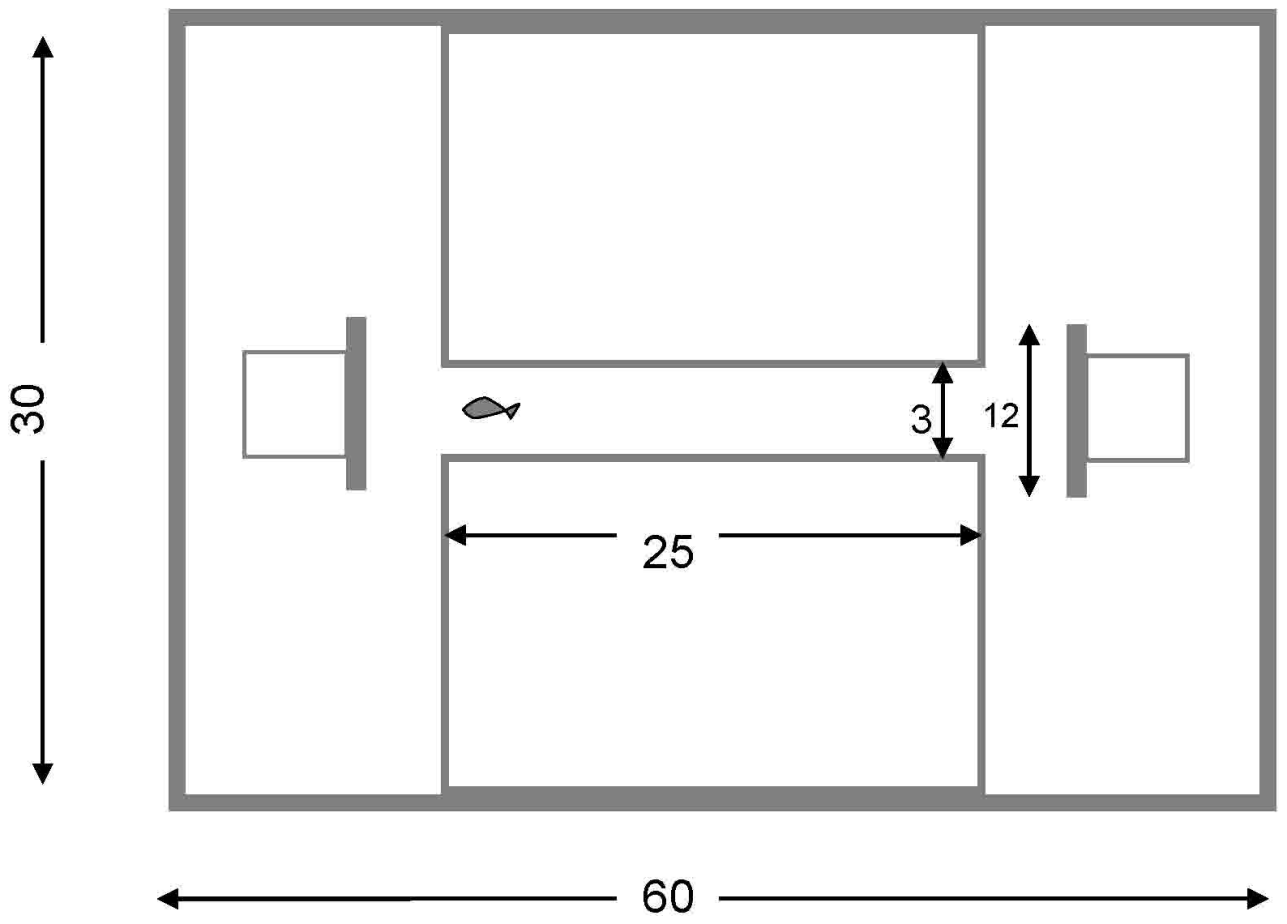
Diagram (top view) of the tank used for the detour test. Measurements are in cm. Not to scale.

Behavioural lateralization was evaluated using a detour test [24]. The detour test is commonly used to evaluate behavioural asymmetries in fish and birds [25], [26]. The apparatus used in this study was based on a design used previously by Bisazza et al. [25] and Dadda et al. [17], and it consists of a two-way T-maze runway which allows to score the direction of the turn (i.e. right or left) of each individual over consecutive runs. Briefly, the experimental apparatus consisted of a glass tank (60×30×40 cm, length×width×height), with a runway in the middle (25×3 cm, length×width) and at both ends of the runway (3 cm ahead of the runway) a white opaque barrier (12×12×1 cm, length×height×width, attached to a 9×9×9 cm glass square behind the barrier) was positioned perpendicular to the orientation of the runway. The runway was delimited by two glass tanks (25×13×20 cm, length×width×height) that provided partitions between the two areas in which the barriers were positioned (Fig. 1). Water in the tank was 4 cm deep. At the start of each trial, a single fish was introduced into the experimental arena and left for 2 min to become accustomed to the environment. During each trial, fish were gently maneuvered to the starting point of the runway. The fish then swam along the runway until it faced the barrier. Fish then had to make a decision to turn left or right around the barrier. Turning was scored by direct observation. The criterion used for scoring was the first turning direction taken by the fish when exiting from the runaway. Ten consecutive runs were observed for each fish, from which the score of the turning direction and the degree of lateralization were obtained. To account for any possible asymmetry in the set up, tests were carried out alternately on the two ends of the runway [25]. Water temperature in the experimental tank was maintained at 26.8 or 29.7°C depending on the treatment. Control water (i.e. not treated with additional CO_2_) was used in all the detour tests. Previous studies [1] have shown that behavioural impairment caused by exposure to elevated CO2 lasts for several days and is not affected by testing fish in CO_2_-treated water versus control water.

A total of 164 individuals were used in the experiments (N = 58 for C- CO_2_/C-Temp; N = 40 for C- CO_2_/E-Temp; N = 42 for the E- CO_2_/C-Temp; and N = 24 for E- CO_2_/E-Temp). In order to compare these 4 groups with respect to their left-right preference in the detour test, we used a relative lateralization index (L*_R_*) according to the following formula [25]: L*_R_* = [(Turn to the right−Turn to the left)/(Turn to the right+Turn to the left)]*100.

Mean L*_R_* was used to assess turning preference (i.e. bias in left or right turns) at the population level. On the basis of the L*_R_* index, individuals were classified between the extreme values of ‘100’ (fish that turned right on all 10 trials) and ‘−100’ (fish that turned left on all 10 trials). A mean L*_R_* near zero indicates that a given sample of the population is neither left- nor right-biased in its turning tendency [15]. The L*_R_* of each group was then compared to a theoretical zero using a one sample t-test [15].

A sample that is not left or right biased may include individuals that are themselves right or left biased. Therefore, the absolute lateralization index (L*_A_*) of each fish was calculated to evaluate the strength of individual lateralization in the detour test irrespective of their preference for right or left turning. The L*_A_* index corresponds to the absolute value of L*_R_*, thus ranging from 0 (an individual that turned in equal proportion to the right and to the left) to 100 (an individual that turned right or left on all 10 trials). L*_A_* thus allowed us to compare the strength of the lateralization (irrespective of its direction) among groups at the individual level. Comparison among the L*_R_* and L*_A_* of all groups was carried out using two-way ANOVAs (with Temperature and CO_2_ as factors) and a Tukey's post-hoc test. Assumptions of normality and homogeneity of variance were examined using residual analysis.

## Results

**Figure 2:**
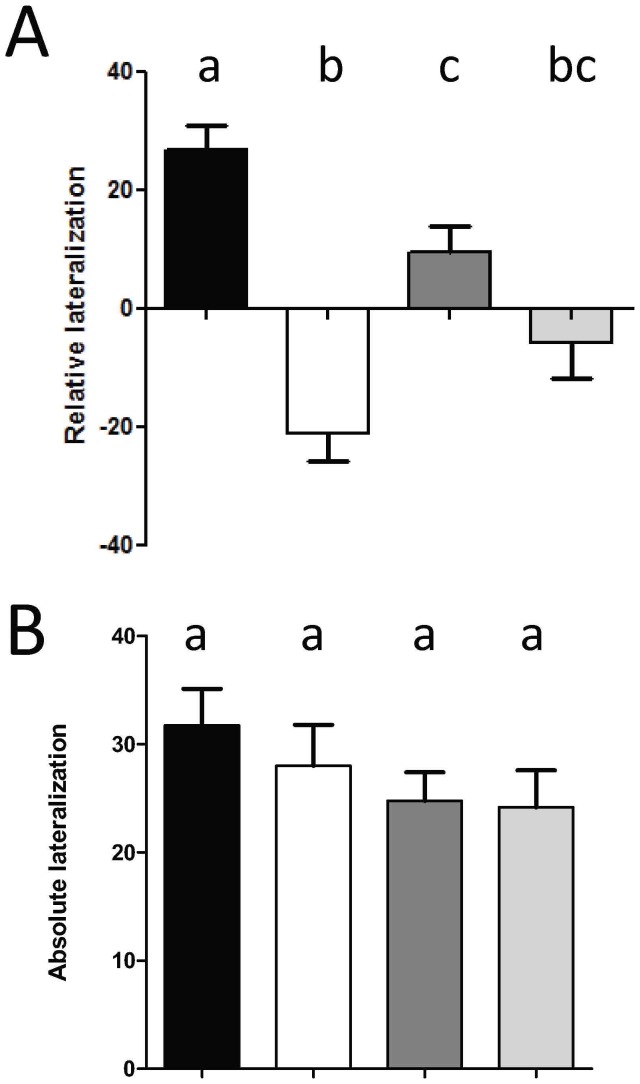
Relative lateralization (A) and absolute lateralization (B) (mean±SE) in each treatment: C-CO_2_/C-Temp (Black bar), E-CO_2_/C-Temp (white bar), C-CO_2_/E-Temp (dark gray bar) and E-CO_2_/E-Temp (light grey bar). Significant differences between treatments (post–hoc test) are indicated by letters.

*L*_R_ values showed a significant interaction between CO_2_ and Temperature (F_1, 160_ = 10.97, P<0.01), and a significant main effect of CO_2_ (F_1, 160_ = 41.45, P<0.001), but not of temperature (F_1, 160_ = 0.05, P>0.5). Post-hoc tests showed significant differences between the Control-CO_2_/Control-Temperature group and all other groups (C-CO_2_/C-Temp *vs.* C-CO_2_/E-Temp, P<0.05; C-CO_2_/C-Temp *vs.* E-CO_2_/C-Temp, P<0.001; C-CO_2_/C-Temp *vs.* E-CO_2_/E-Temp, P<0.001), and between the Control-CO_2_/Elevated-Temperature and the Elevated-CO_2_/Control-Temperature group (P<0.001) (Fig. 2A). *L*_A_ was not affected by CO_2_, temperature or an interaction between these factors (F_1, 160_ = 0.36; F_1, 160_ = 2.22; F_1, 160_ = 0.19, respectively, P>0.1 in all cases; Fig. 2B).

A bias in the relative lateralization index was found at the population level for Control-CO_2_/Control-Temperature, Control-CO_2_/Elevated-Temperature and Elevated-CO_2_/Control-Temperature [one sample t-tests (supplementary material): C-CO_2_/C-Temp, t_(57)_ = 6.65, P<0.0001; C-CO_2_/E-Temp, t_(39)_ = 4.37, P<0.0001; E-CO_2_/C-Temp, t_(41)_ = 2.15, P<0.05]. However, elevated CO_2_ influenced the direction of lateralization; individuals from Control-CO_2_/Control-Temperature and Control-CO_2_/Elevated-Temperature had a significant preference for right turns (*L*_R_ = 26.9±4.04 and 9.5±4.43, respectively), whereas individuals from Elevated-CO_2_/Control-Temperature had a significance preference for left turns (*L*_R_ = −21.0±4.80). Individuals from the Elevated-CO_2_/Elevated-Temperature group did not show a significant directional bias, with their *L*_R_ being not significantly different from zero (*L*_R_ = −5.8±5.96; t_(23)_ = 0.98, P>0.1).

**Figure 3:**
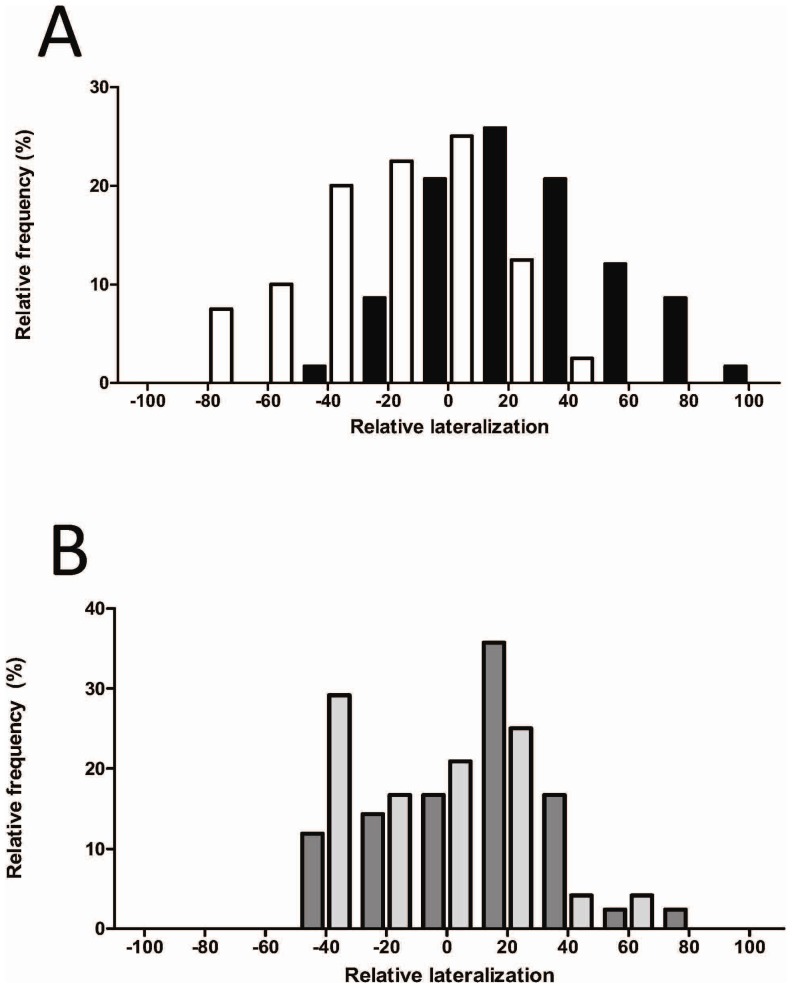
Relative frequency distributions of *L*_R_ in C-CO_2_/C-Temp (black bars) and E-CO_2_/C-Temp (white bars) (A) and in C-CO_2_/E-Temp (dark grey bars) and E-CO_2_/E-Temp (light grey bars) (B). Positive and negative values indicate right and left turns, respectively. The extreme values of |100| indicate fish that turned in the same direction on all 10 trials.

Therefore, elevated CO_2_ reversed the relative lateralization bias from right to left, as indicated by the mirror images of the directionally-biased *L*_R_ frequency distributions of the Control-CO_2_/Control-Temperature and Elevated-CO_2_/Control-Temperature treatments (Fig. 3A). The high-temperature treatment attenuated this effect and the two high-temperature distributions (C-CO_2_/E-Temp and E-CO_2_/E-Temp) largely overlapped (Fig. 3B).

## Discussion

Our work is the first one to test the effect of exposure to elevated CO_2_ levels in a species that is lateralized at the population level. Previous work, carried out in species that were unbiased at the population level [7], [27], show that lateralization at the individual level is disrupted by elevated CO_2._ Here we show that the turning direction of a species lateralized at the population level can be reversed (from right to left), with interesting implications for brain control mechanisms (see below). Furthermore, we also tested the modulating effect of temperature, and we found a significant interaction with elevated temperature, such that individuals exposed to higher temperatures showed a reduced level of relative lateralization.

Although temperature is known to have a fundamental effect on fish metabolism, activity and performance [13], little is known about its effect on behavioural tasks. While temperature has been shown to affect some behavioural decisions [26], no previous studies have tested its effect on lateralization, or how it may interact with other environmental variables to affect lateralization. Here, we show that exposure to increased temperature has a dampening effect on the lateralization level of the population, in both control and elevated CO_2_ treatments. This indicates a malfunction or attenuation of the turning bias as a result of elevated temperature, the mechanism of which may be related to temperature effects on the cerebral processes at the basis of the left/right choice. Although the elevated temperature used in our experiment was 3°C higher than the ambient October temperature of 27°C, *P. wardi* do experience temperatures around 30°C in the middle of summer. Therefore, it would be interesting to test if the attenuation of lateralization is due to the unseasonal increase in temperature, or to the high temperature *per se*, regardless of season/acclimation time.

The shift in right to left lateralization under elevated CO_2_ suggests that the altered neurotransmitter function responsible for behavioural impairment in marine fishes may generate an inversion of the functional brain asymmetry that is the basis of behavioural lateralization, as shown in *Neopomacentrus azysron* [12]. A possible interpretation of these results is that the reversal from right turning bias when facing the opaque barrier (suggesting left brain control, [28]) to left-turning bias (suggesting right brain control) is a consequence of stress. Previous work indeed suggests that stressed animals tend to rely on the predominant use of the right hemisphere [29]. Interestingly, the species we tested here shows a relatively low level of individual lateralization (*L*_A_ around 30), compared to *N. azysron* (*L*_A_ around 50) [6]. This suggests that the precise mechanism responsible for individual lateralization may differ between the two species, which could explain why individual behavioral lateralization was disrupted in *N. azysron* [6] while it was reversed in *P. wardi*.

While CO_2_ does not affect the individual lateralization level (i.e. *L*_A_) of *P. wardi*, its effect of reversing the side bias from right to left might still have ecological implications. Population-level lateralization has been suggested to be more likely in gregarious species, because of the need to maintain group cohesion [14]. *P. wardi* is not gregarious during the juvenile phase [27] and its population-level lateralization confirms that this pattern can also occur in non-gregarious individuals [15]. Although the evolutionary pressure that produces such pattern of lateralization in solitary species is unknown, it may be related to asymmetries in their natural environment, e.g. in their predators [14]. Alternatively, the population-level lateralization observed in juveniles may be the result of a carry-over effect of shoaling during the larval phase which precedes the juvenile stage only by a few days. If we assume that the *L*_R_ pattern of distribution found in populations caught in the field is the result of natural selection, the decrease of *L*_R_ caused by temperature is likely to diminish any advantage provided by the population-level directional bias. Similarly, side-shifts in the direction bias as a result of environmental changes, such as elevated CO_2_, could decrease the performance of the population.

Our results provide strong evidence that the combined effect of elevated CO_2_ and temperature can vary both the direction and the magnitude of lateralization in fish. Because lateralized individuals show superior performance in anti-predator responses [17], gregariousness [16], and multitasking such as foraging while performing predator vigilance [18], it is likely that the combined effect of CO_2_ and temperature will interfere with the natural behaviour of fish and this may have consequences for the outcome of important ecological interactions, such as predator-prey encounters.
